# Genetic Variants Masking Functional Neurological Disorder: A Case of Gait Disturbance with SGCD and MTHFR Heterozygosity

**DOI:** 10.1192/j.eurpsy.2026.10733

**Published:** 2026-07-31

**Authors:** A. Uzturk, S. Deveci, F. Azman Iste, E. Suleymanli, A. Koksal

**Affiliations:** 1 Department of Psychiatry; 2 Department of Neurology, Basaksehir Cam And Sakura City Hospital, Istanbul, Türkiye

## Abstract

**Introduction:**

Functional Neurological Disorder (FND) is characterized by genuine neurological symptoms without structural explanation, often mimicking neuromuscular disease(Espay et al., 2018; Perez et al., 2021). Sarcoglycan Delta (SGCD) mutations cause limb-girdle muscular dystrophy type 2F (LGMD2F) in homozygous/compound heterozygous states, while heterozygotes are usually asymptomatic (Gordon et al., 2009). In contrast, Methylenetetrahydrofolate Reductase (MTHFR) polymorphisms are strongly associated with psychiatric vulnerability—including depression, schizophrenia, and bipolar disorder(Lewis et al., 2006; Meng et al., 2022)—suggesting predisposition for functional symptoms.

**Objectives:**

To highlight a case in which a 5-year history of gait disturbance was initially masked by incidental genetic findings, and to show how repeated examinations enabled FND diagnosis. The case underscores how functional symptoms affect electrophysiology, the need for psychiatric support, and challenges from genetic variants.

**Methods:**

Comprehensive neurological, psychiatric, and genetic evaluations were performed.

**Results:**

A 48-year-old woman presented with a 5-year history of fluctuating gait disturbance, initially suspected as LGMD. Genetic testing revealed heterozygous SGCD and MTHFR variants. Neurological examination results varied: sometimes spastic and weak, sometimes (especially when distracted) completely normal. EMG performed during outpatient follow-up showed mild, non-specific proximal myopathic changes, whereas a repeat EMG during hospitalization was completely normal. Brain and spinal MRI were unremarkable, and antibody panels were negative. Following lumbar puncture, neurological symptoms spontaneously resolved. Psychiatric assessment revealed anxiety with preserved insight and judgment. Psychoeducation and psychotherapy were recommended.

**Conclusions:**

This case demonstrates the diagnostic complexity when genetic findings suggest neuromuscular pathology but functional mechanisms predominate. SGCD heterozygosity represents a carrier state without overt disease, while MTHFR variants may increase psychiatric vulnerability and influence FND presentation (Gordon et al., 2009; Lewis et al., 2006; Meng et al., 2022). The patient’s 5-year fluctuating course, inconsistency between clinical examination and functional capacity, normal neuroimaging and electrophysiology, and transient improvement after lumbar puncture supported FND over neuromuscular disease (Espay et al., 2018; Perez et al., 2021). Recognizing positive diagnostic features—variability, reversibility, and internal inconsistency—is essential, as overinterpretation of genetic findings can lead to misdiagnosis, unnecessary interventions, and delayed psychiatric care. Multidisciplinary evaluation and timely psychiatric intervention are essential for accurate diagnosis.

**Disclosure of Interest:**

None Declared

